# Impact of the COVID pandemic on mental health and training opportunities of Public Health Residents from 4 European countries: A cross-sectional study

**DOI:** 10.3389/fpubh.2023.1044171

**Published:** 2023-03-07

**Authors:** Giovanna Failla, Marta Caminiti, José Chen-Xu, Giuseppina Lo Moro, Nausicaa Berselli, Madalena Cabral Ferreira, Filipa Malcata, David Peyre-Costa, Roberto Croci, Giorgia Soldà, Angelo Capodici, Caterina Morcavallo, Francesco Traglia, Fabrizio Cedrone, Ilaria Storti, Alfonso Alonso Jaquete, Martina Antinozzi, Anca Vasiliu

**Affiliations:** ^1^Department of Life Sciences and Public Health, Catholic University of the Sacred Heart, Rome, Italy; ^2^Department of Public Health Sciences, University of Perugia, Perugia, Italy; ^3^Public Health Unit, Primary Health Care Cluster Baixo Mondego, Coimbra, Portugal; ^4^Department of Public Health Sciences, University of Turin, Turin, Italy; ^5^Department of Biomedical, Metabolic and Neural Sciences, University of Modena and Reggio Emilia, Modena, Italy; ^6^Public Health Unit, Primary Health Care Cluster Pinhal Litoral, Leiria, Portugal; ^7^Public Health Unit, Primary Health Care Cluster Porto Ocidental, Porto, Portugal; ^8^Public Health Unit, University Hospital of Montpellier, Montpellier, France; ^9^School of Medicine, University Vita-Salute San Raffaele, Milan, Italy; ^10^School of Hygiene and Preventive Medicine, Department of Biomedical and Neuromotor Sciences, Public Health and Medical Statistics, University of Bologna, Bologna, Italy; ^11^Department of Biomedical Sciences and Human Oncology, School of Medicine, University of Bari Aldo Moro, Bari, Italy; ^12^Department of Biomedicine and Prevention, University of Rome Tor Vergata, Rome, Italy; ^13^Health Management of “SS. Spirito” Hospital of Pescara, Local Health Authority of Pescara, Pescara, Italy; ^14^Department of Cardiothoracic and Vascular Sciences and Public Health, University of Padua, Padua, Italy; ^15^Preventive Medicine and Public Health Unit, Health Department of the Government of Cantabria, Santander, Spain; ^16^Sapienza University of Rome, Rome, Italy; ^17^Department of Pediatrics, Global Tuberculosis Program, Baylor College of Medicine, Houston, TX, United States

**Keywords:** public mental health, healthcare workforce, Europe, pandemic, medical residents

## Abstract

**Objectives:**

There is little evidence on the impact of the COVID-19 pandemic on Public Health Residents' (PHR) mental health (MH). This study aims at assessing prevalence and risk factors for depression, anxiety and stress in European PHR during the COVID-19 pandemic.

**Methods:**

Between March and April 2021, an online survey was administered to PHR from France, Italy, Portugal and Spain. The survey assessed COVID-19 related changes in working conditions, training opportunities and evaluated MH outcomes using the Depression Anxiety Stress Scales-21. Multivariable logistic regressions were applied to identify risk factors.

**Results:**

Among the 443 respondents, many showed symptoms of depression (60.5%), anxiety (43.1%) and stress (61.2%). The main outcome predictors were: female gender for depression (adjOR = 1.59, 95%CI [1.05–2.42]), anxiety (adjOR = 2.03, 95%CI [1.33–3.08]), and stress (adjOR = 2.35, 95%CI [1.53–3.61]); loss of research opportunities for anxiety (adjOR = 1.94, 95%CI [1.28–2.93]) and stress (adjOR = 1.98, 95%CI [1.26–3.11]); and COVID-19 impact on training (adjOR = 1.78, 95%CI [1.12–2.80]) for depression.

**Conclusions:**

The pandemic had a significant impact on PHR in terms of depression, anxiety and stress, especially for women and who lost work-related opportunities. Training programs should offer PHR appropriate MH support and training opportunities.

## Introduction

The COVID-19 pandemic has inflicted a heavy burden on many national healthcare systems, as well as on the global health workforce. Throughout Europe, fragmented and uneven policies have been experimented to hire or redeploy health workers in order to create adequate “surge capacity” (1). During the pandemic, the massive influx of patients, reinforced health protocols, and lack of proper equipment, represented additional constraints for all healthcare workers (HWs) (1). This situation was often accompanied by an increased workload leading to compassion fatigue, through experiences of stress or trauma (2). HWs experienced situations of acute stress, even PTSD, which led to considerable consequences, like burnout and adverse mental health outcomes (3–5). Moreover, many studies conducted on physicians during the COVID-19 pandemic showed that female doctors were more likely to suffer from depression, anxiety, stress and burnout, and they were associated with worse mental health outcomes (6–10).

In order to fill in the human resource gap and add to the available capacity, public health practitioners have taken the role of frontline HWs in the COVID-19 response in many European countries (11, 12). Public Health is a postgraduate speciality for medical doctors in several European countries, with a training length of 4 years in France, Italy, Portugal and Spain. Public Health Residents (PHR) represent a substantial proportion of the public health workforce in some European countries. PHR training puts emphasis on a population health approach that should meet public health practice needs and support public health workforce development (13).

Although Western European countries benefit from high-performance health systems, the COVID-19 has severely challenged them. A study that aimed at analyzing the efficiency of the health systems of 31 European countries in treating COVID-19 affirmed that Portugal's health system had an average efficacy, whereas France, Italy and Spain's health systems had a low efficiency (14). The management of the pandemic had an impact on residents' medical training. For example, a study of the European Society of Residents in Urology found that in Italy and Spain, residents' training has been made through online smart-learning circuits, webinars, and video calls. In France, residents who had fellowships planned locally or abroad were unable to attend and hospital rotations have been postponed (15). The data on the reduction of residents' training capacity in Europe is consistent taking among different medical speciality, such as neurology, otorhinolaryngology, pediatric surgery, plastic surgery (16–19).

Prior to the pandemic, a meta-analysis investigating the mental health of medical residents of different specialties showed a significant increase of depressive symptoms among trainees within a year of beginning training and a similar prevalence of depressive symptoms across specialities and countries (20). These findings confirm that mental distress among physicians during medical training is quite common, even outside the period of pandemic alertness and response. COVID-19 has strikingly changed the lives and outlook of PHR in a very short time, and residency programmers have been heavily affected. Some PHR have performed clinical duties by being deployed to direct patient care positions. Most prominently, they have helped in the implementation of containment and mitigation measures by participating in activities such as “testing and tracing,” enforcing hospital-based preventive protocols, and administering COVID-19 vaccinations.

Undoubtedly, being a public health resident during a global pandemic can bring valuable training opportunities, nevertheless, COVID-19 related work risks to overtake routine practical and theoretical training. In addition, non-COVID-19 related opportunities are scarcer than before the pandemic, and this could compromise the acquisition of the complex set of skills needed for a public health specialist (21).

The lack of awareness of mental health issues among HW brings many consequences, which can ultimately result in burnout (22, 23). The importance of a strong social support system for HW, especially during the first years of working as a resident, is vital to the adaptation to the new context and learning to have access to social support and make use of it in order to prevent emotional exhaustion. HW who are less inclined to seek social support should receive extra attention, as well as those with increased risk of secondary traumatisation (24).

This study is part of a research project carried out by the European Network of Medical Residents in Public Health (25). The underlying hypothesis of this work is that COVID-19 had a detrimental impact on the mental health, and training opportunities of PHR in Europe. As European countries had different responses to the pandemic, this is a non-exhaustive comparison. Nevertheless, to our knowledge, no other study has looked into the impact of the COVID-19 pandemic in this specific population. The main objective of this study is to assess the proportion and risk factors of pandemic-related depression (D), anxiety (A), and stress (S) in the population of Public Health residents from France, Italy, Portugal and Spain. The secondary objective of this work is to assess the impact of COVID-19 on their training opportunities.

## Methods

### Study design and population

A cross-sectional study was conducted between March 22nd and April 12th 2021 using an online questionnaire addressed to the PHR of four different European countries: France, Italy, Portugal and Spain.

The study population consisted of all PHR in training during the study period and a total of 2010 PHR between four countries: France (*n* = 320), Italy (*n* = 1,180), Portugal (*n* = 210), and Spain (*n* = 300).

### Data collection tool

The questionnaire was distributed through the networks of national public health residents' associations and was composed of two sections: (1) general characteristics and training opportunities, and (2) mental health assessment. The full questionnaire is available in the Supplementary material.

#### Section 1: General characteristics and training opportunities

The information collected through the questionnaire concerned six initial questions about socio-demographic data such as age, year of residency, gender (binary, other), living arrangement (alone, with family/friends/partner/other) and relationship status (single/in a couple/married/divorced/widowed/other).

Other five questions investigated whether the COVID-19 pandemic had an impact on the educational path in terms of research opportunities and professional training, and whether PHR were involved (directly or indirectly) in COVID-19 related tasks. Four questions had a dichotomous yes/no answer and one question had four different answer options.

The first section was translated in the four languages of the target countries: French, Italian, Spanish and Portuguese, in order to increase the response rate by facilitating the understanding of the questions.

#### Section 2: Mental health assessment

*For the second section of the questionnaire*, we used the revised Depression Anxiety Stress Scale Short Version (DASS-21) composed of 21 items (26), to investigate symptoms related to events that happened during the COVID-19 pandemic the year before the data collection. This shortened validated scale shows good psychometric properties whilst being less time-consuming compared to the version composed of 42 items (27). We used the validated translation of the scale in the four study languages in order to ensure correct terminology was used for each language (28–31).

The DASS-21 investigates three constructs: depression, anxiety and stress, each with seven items. Each item is scored on a 4-point Likert scale (0–3). The score of each axis is calculated by summing the scores of the seven items and then multiplying by 2 to lie within a 0–42 scale. A higher score indicates more severe levels of distress.

Depression concerns dysphoria, despair, life depreciation, lack of interest/involvement, anhedonia and inertia; anxiety relates to arousal of the autonomic nervous system, effects on skeletal muscles, situational anxiety and subjective experience of anxious affects; stress is related to the presence of chronic non-specific arousal levels, relaxation difficulties, nervous excitement, irritability, agitation, hyperactivity, impatience. The severity of the subscale scores can be categorized as follows: normal (depression: 0–9; anxiety: 0–7; stress: 0–14), mild (depression: 10–13; anxiety: 8–9; stress: 15–18), moderate (depression: 14–20; anxiety: 10–14; stress: 19–25), severe (depression: 21–27; anxiety: 15–19; stress: 26–33), extremely severe (depression: ≥28; anxiety: ≥20; stress: ≥34) (27).

### Statistical analysis

Descriptive analyses were carried out for all variables. Categorical variables were expressed as frequencies and percentages. Continuous variables were expressed as median and interquartile range (IQR).

DASS-21 has three binary outcomes: depression, anxiety, and stress. We considered the cut-off score of the DASS-21 as follows: for depression >9, for anxiety >7, and for stress >14 (27).

Cronbach's α was calculated to test the internal consistency of depression, anxiety, and stress scales in the sample. Chi-squared tests were computed to assess differences between the groups defined by the outcomes (for age: Mann Whitney *U*-test).

The binary outcomes of depression, anxiety and stress were used as dependent variables in univariable regressions for all the three outcomes [results expressed as odds ratios (OR) and their 95% Confidence Interval (CI)]. In addition, for each outcome, a multivariable logistic regression model was performed [results expressed as adjusted odds ratios (adjOR), 95% CI].

The independent variables age and gender were entered in each model. Then, to select the other independent variables to be included in the final model, a backward elimination method was used for each outcome. SPSS (version 27) was used, and a two-tailed *p*-value < 0.050 was considered statistically significant for all analyses. Missing values were excluded.

### Ethical considerations

This study was conducted in conformity with the Declaration of Helsinki. An electronic informed consent was obtained from each participant before the start of the investigation. All subjects agreed to the processing of their anonymous personal data. In conducting our survey, we respected two important ethical issues: confidentiality and informed consent. The respondent's right to confidentiality was respected, data have been anonymised, in absolute compliance with legal requirements on data protection (GDPR, Recital 162).

## Results

A total of 445 PHR took the survey. Among them, two residents refused for their data to be used, thus these two records were deleted. Therefore, we analyzed 443 responses. Our sample represented 22.0% of the total number of 2010 potentially eligible PHR (i.e., the total number of PHR in the participating countries). Out of the total of Public Health Residents of each country, Italy's responses amounted to 51% (*n* = 226), Portugal's 19.6% (*n* = 87), Spain's 16.7% (*n* = 74), and France's 12.6% (*n* = 56). The study sample consisted of 61.4% PHR identifying as females (*n* = 272) and 37.5% identifying as males (*n* = 166). The remaining 1.1% identified with neither or preferred not to declare gender (*n* = 5).

### General characteristics

More than half respondents were in the first 2 years of residency (*n* = 295, 66.6%), with the first-year capping at 39.7% (*n* = 176), and second-year residents representing 26.9% of the total (*n* = 119). Third, fourth, fifth and sixth year represented 16.5 (*n* = 73), 16.3 (*n* = 72), 0.5 (*n* = 2), and 0.2% (*n* = 1) respectively. Most of the interviewed demographic was living with family or a partner (*n* = 270, 60.9%), 24.4% were living alone (*n* = 108) and 12.4% with friends (*n* = 55), the remaining 2.3% did not respond (*n* = 10). A total of 65% of respondents were either married (*n* = 65) or in a relationship (*n* = 223), and 31.8% were single (*n* = 141).

In considering frontline activities in the COVID-19 pandemic, 10.8% of respondents had direct contact with established cases (*n* = 48), while the majority (*n* = 269, 60.7%) did not have contact with confirmed COVID-19 patients even though they were involved in pandemic related activities (Table 1).

**Table 1:** Characteristics of the study population (Public Health Residents' Mental Health, Europe, 2021–2022).

**Variable**	**Frequency**
**Country**	
France	56 (12.6%)
Italy	226 (51.0%)
Portugal	87 (19.6%)
Spain	74 (16.7%)
Age (median, interquartile range)	29.0 (4)
**Gender**	
Female	272 (61.4%)
Male	166 (37.5%)
Other/prefer not to say	5 (1.1%)
**Living arrangement**	
Alone	108 (24.4%)
Family/partner	270 (60.9%)
Friends	55 (12.4%)
Prefer not to say	10 (2.3%)
**Year of residency**	
1st	176 (39.7%)
2nd	119 (26.9%)
3rd	73 (16.5%)
4th or higher	75 (16.9%)
**Relationship status**	
Single	141 (31.8%)
In a relationship	223 (50.3%)
Married	65 (14.7%)
Divorced	5 (1.1%)
Prefer not to say	9 (2.0%)
Work in a COVID-19 related project	263 (59.4%)
Less research opportunities during the pandemic	159 (35.9%)
Impacted on the Public Health Residency	339 (76.5%)
**Work in the frontline**	
A. No COVID-19 related activities	87 (19.6%)
B. COVID-19 related activities with or without direct contact with patients	269 (60.7%)
C. Direct contact with COVID-19 patients without COVID-19 related activities	48 (10.8%)
D. Direct contact + COVID-19 related activities	38 (8.6%)

The remaining characteristics of the population are available in Table 1.

### Training and research opportunities

While 59.4% of participants worked on a COVID-19-related project, 35.9% reported to have found less research opportunities. Moreover, 76.5% of respondents reported to have had their training in public health impacted in some way.

### DASS-21 descriptive analyses

**Figure 1:**
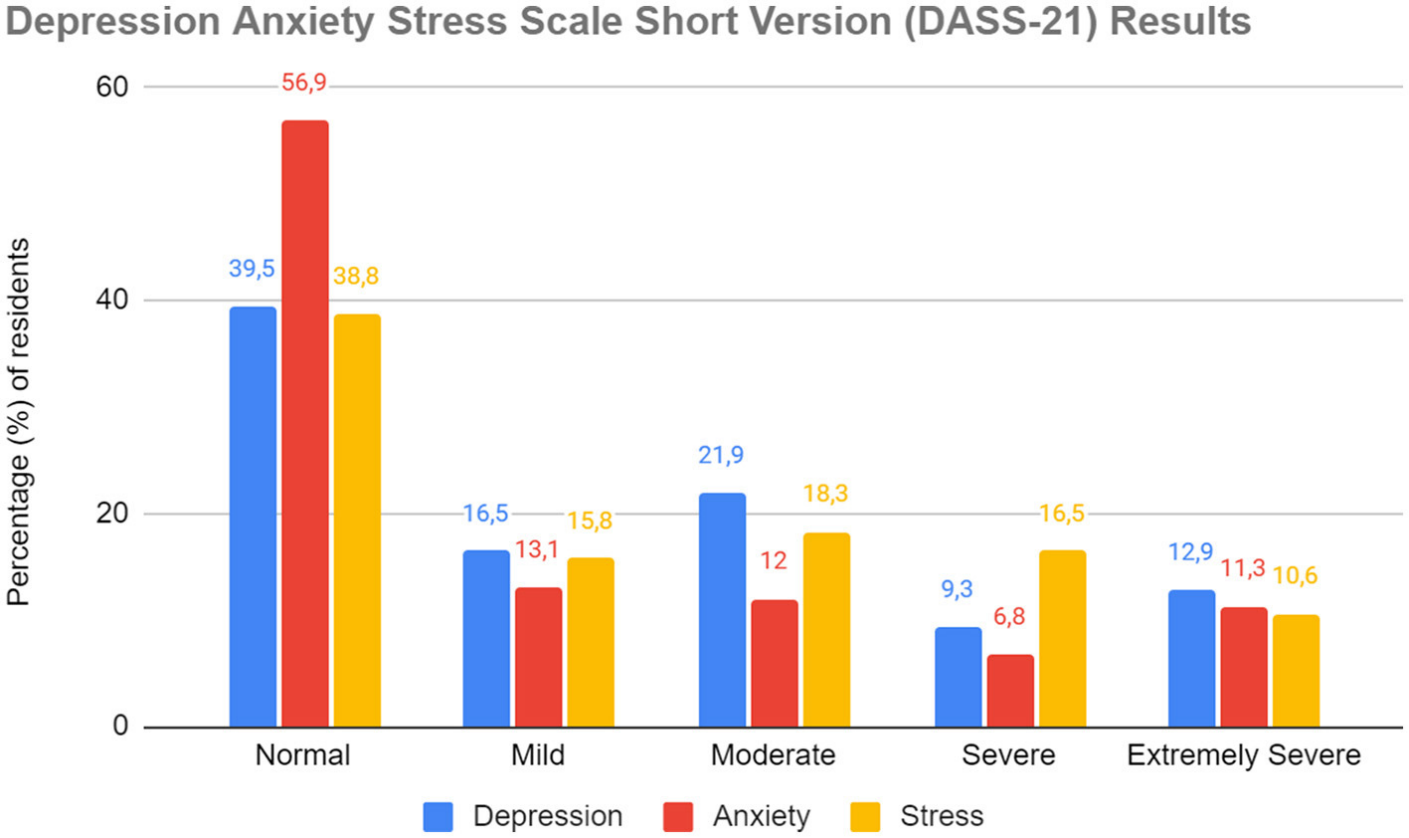
Percentage of subjects affected by different levels of depression, anxiety and stress, according to the three subscales of the test Depression Anxiety Stress Scale short version, sorted according to the growing severity of the symptoms found (from “Normal” to “Extremely Severe”) (Public Health Residents' Mental Health, Europe, 2021–2022).

As shown in Figure 1, DASS-21 scores revealed a large number of residents reporting symptoms of depression, anxiety, and stress on a mild to extremely severe scale. Specifically, 60.6% of the sample showed depressive symptoms, 43.2% of the participants reported anxiety symptoms (*n* = 191) and 61.2% of the interviewed residents experienced stress (*n* = 271). Many subjects exhibited these traits to a severe or extremely severe degree: 22.2% for depressive symptoms (*n* = 98), 18.1% for anxiety symptoms (*n* = 80), 27.1% for stress (*n* = 120). The median score was 12 (IQR = 4–20) for the depression subscale, 6 (IQR = 2–12) for the anxiety subscale, and 18 (IQR = 12–26) for the stress subscale. In this sample, Cronbach's α was 0.913 for the depression subscale, 0.838 for the anxiety subscale, and 0.892 for the stress subscale.

### Depression

Bivariate regressions with the presence of depressive symptoms as outcome showed significant associations: participants who thought their Public Health training was impacted by the pandemic (OR 1.95, 95% CI 1.25–3.03, *p* = 0.003) or were female (OR 1.56, 95% CI 1.05–2.31, *p* = 0.026) had a higher probability of experiencing depression.

The final multivariable model confirmed the statistical significance of the relationships between depressive symptoms and female participants (adjOR 1.62, 95% CI 1.06–2.48, *p* = 0.025) and people who considered their Public eHHealth training was impacted by the pandemic (adjOR 1.74, 95% CI 1.09–2.76, *p* = 0.020) (Table 2).

**Table 2:** Factors associated with depression, resulting from the bivariate and multivariate regression analysis of the data (Public Health Residents' Mental Health, Europe, 2021–2022).

**Variable**	**Bivariate regression OR [95% CI]**	***p*-value**	**Multivariate regression adjOR [95% CI]**	***p*-value**
Age	0.98 [0.94–1.02]	0.388	0.98 [0.93–1.02]	0.280
Gender (female:male)	1.56 [1.05–2.31]	0.026	1.62 [1.06–2.48]	0.025
**Living arrangement**				
Alone (reference)	1.0			
Family/partner	0.72 [0.45–1.14]	0.165		
Friends	1.03 [0.52–2.03]	0.935		
Prefer not to say	2.17 [0.44–10.74]	0.342		
**Year of residency**				
1st (reference)	1.0			
2nd	1.24 [0.77–1.99]	0.383		
3rd	0.93 [0.54–1.61]	0.795		
4th or higher	1.45 [0.82–2.56]	0.197		
**Relationship status**				
Single (reference)	1.0			
In a relationship	0.73 [0.47–1.13]	0.160		
Married	0.70 [0.39–1.28]	0.252		
Divorced	0.36 [0.06–2.20]	0.265		
Prefer not to say	4.26 [0.52–35.06]	0.178		
Work in a COVID-19 related project	1.04 [0.71–1.54]	0.823		
Less research opportunities during the pandemic	1.44 [0.96–2.16]	0.075		
Impacted on the PH Residency	1.95 [1.25–3.03]	0.003	1.74 [1.09–2.76]	0.020
**Work in the frontline**				
No COVID-19 related activities (reference)	1.0			
COVID-19 related activities	1.44 [0.88–2.34]	0.145		
Direct contact with COVID-19 patients without COVID-19 related activities	1.30 [0.64–2.66]	0.474		
Direct contact + COVID-19 related activities	1.17 [0.54–2.53]	0.689		

OR, odd ratio; adjOR, adjusted odd ratio; CI, confidence interval; PH, public health.

### Stress

Getting less research opportunities due to the pandemic (OR 2.25, 95% CI 1.47–3.42, *p* < 0.001), being in the second year (OR 2.12, 95% CI 1.30–3.45, *p* = 0.003) or in the last year of residency (OR 2.46, 95% CI 1.37–4.41, *p* = 0.003), and the perception of their Public Health training being impacted by the pandemic (OR 2.02, 95% CI 1.30–3.16, *p* = 0.002) had a higher chance of increasing stress.

Moreover, the association with gender impacted the outcome, with female participants (OR 2.18, 95% CI 1.46–3.24, *p* < 0.001) more likely to experience stress. The multivariate regression highlighted these associations between stress and female gender (adjOR 2.35, 95% CI 1.53–3.61, *p* < 0.001), fewer research opportunities (adjOR 1.98, 95% CI 1.26–3.11, *p* = 0.003), and year of residency, specifically with higher likelihood of stress in the second year (adjOR 1.77, 95% CI 1.06–2.95, *p* = 0.029) and last year of residency (adjOR 2.30, 95% CI 1.20–4.38, *p* = 0.012) (Table 3).

**Table 3:** Factors associated with stress, resulting from the bivariate and multivariate regression analysis of the data (Public Health Residents' Mental Health, Europe, 2021–2022).

**Variable**	**Bivariate regression OR [95% CI]**	***p*-value**	**Multivariate regression adjOR [95% CI]**	***p*-value**
Age	1.00 [0.96–1.05]	0.930	0.96 [0.92–1.01]	0.116
Gender (female:male)	2.18 [1.46–3.24]	<0.001	2.35 [1.53–3.61]	<0.001
**Living arrangement**				
Alone (reference)	1.0			
Family/partner	1.12 [0.71–1.77]	0.618		
Friends	1.25 [0.64–2.44]	0.514		
Prefer not to say	2.86 [0.58–14.10]	0.197		
**Year of residency**				
1st (reference)	1.0			
2nd	2.12 [1.30–3.45]	0.003	1.77 [1.06–2.95]	0.029
3rd	1.54 [0.88–2.68]	0.131	1.49 [0.82–2.70]	0.186
4th or higher	2.46 [1.37–4.41]	0.003	2.30 [1.20–4.38]	0.012
**Relationship status**				
Single (reference)	1.0			
In a relationship	1.02 [0.66–1.57]	0.922		
Married	1.33 [0.72–2.45]	0.367		
Divorced	0.45 [0.07–2.79]	0.393		
Prefer not to say	5.43 [0.66–44.59]	0.115		
Work in a COVID-19 related project	1.19 [0.80–1.75]	0.388		
Less research opportunities during the pandemic	2.25 [1.47–3.42]	<0.001	1.98 [1.26–3.11]	0.003
Impacted on the PH Residency	2.02 [1.30–3.16]	0.002		
**Work in the frontline**				
No COVID-19 related activities (reference)	1.0			
COVID-19 related activities	1.16 [0.70–1.90]	0.566		
Direct contact with COVID-19 patients without COVID-19 related activities	0.67 [0.33–1.37]	0.274		
Direct contact + COVID-19 related activities	1.15 [0.53–2.53]	0.721		

OR, odd ratio; adjOR, adjusted odd ratio; CI, confidence interval; PH, public health.

### Anxiety

The bivariate regression identified significant associations between increased likelihood of reporting anxiety symptoms and being a female resident (OR 1.88, 95% CI 1.26–2.08, *p* = 0.002) or a PHR who got less research opportunities due to the pandemic (OR 1.93, 95% CI 1.30–2.86, *p* = 0.001). The final multivariate regression confirmed the associations between anxiety symptoms and female participants (adjOR 2.03, 95% CI 1.33–3.08, *p* = 0.001), and participants who had less research opportunities due to COVID-19 (adjOR 1.94, 95% CI 1.28–2.93, *p* = 0.002) (Table 4).

**Table 4:** Factors associated with anxiety, resulting from the univariate and multivariate regression analysis of the data (Public Health Residents' Mental Health, Europe, 2021–2022).

**Variable**	**Bivariate regression OR [95% CI]**	***p*-value**	**Multivariate regression adjOR [95% CI]**	***p*-value**
Age	0.98 [0.94–1.02]	0.343	0.95 [0.91–1.00]	0.051
Gender (female: male)	1.88 [1.26–2.08]	0.002	2.03 [1.33–3.08]	0.001
**Living arrangement**				
Alone (reference)	1.0			
Family/partner	1.10 [0.70–1.73]	0.670		
Friends	1.17 [0.61–2.24]	0.644		
Prefer not to say	0.35 [0.07–1.73]	0.197		
**Year of residency**				
1st (reference)	1.0			
2nd	1.25 [0.78–2.00]	0.343		
3rd	0.78 [0.44–1.37]	0.390		
4th or higher	1.30 [0.76–2.24]	0.340		
**Relationship status**				
Single (reference)	1.0			
In a relationship	0.96 [0.62–1.47]	0.845		
Married	1.62 [0.90–2.93]	0.109		
Divorced	0.93 [0.15–5.72]	0.935		
Prefer not to say	1.11 [0.29–4.32]	0.878		
Work in a COVID-19 related project	1.16 [0.79–1.71]	0.440		
Less research opportunities during the pandemic	1.93 [1.30–2.86]	0.001	1.94 [1.28–2.93]	0.002
Impacted on the PH Residency	1.28 [0.82–2.01]	0.274		
**Work in the frontline**				
No COVID-19 related activities (reference)	1.0			
COVID-19 related activities	1.07 [0.66–1.75]	0.780		
Direct contact with COVID-19 patients without COVID-19 related activities	0.74 [0.36–1.54]	0.420		
Direct contact + COVID-19 related activities	1.22 [0.57–2.62]	0.616		

OR, odd ratio; adjOR, adjusted odd ratio; CI, confidence interval; PH, public health.

## Discussion

This study aimed to address the impact of the COVID-19 pandemic in mental health outcomes among PHR.

Overall, the main risk factors in our study associated with negative mental health psychometric outcomes were PH training challenges, loss of training opportunities, residency seniority and female gender.

### Training and research opportunities

According to the findings presented in this study, residents who thought their Public Health training was impacted by COVID-19 had a higher chance of increased stress and depression prevalence. During the pandemic, traditional residents' education has been compromised due to the disruption in training, which can lead to long-term detrimental consequences (32). Occasional and not uniform use of remote education platforms among residents led to transit from an in-person training to an online-training, trying to build connections between residents and teachers where possible. However, this strategy did not minimize mental health risks in the study population (33).

In the current study, participants who got less research opportunities due to the pandemic had greater likelihood of reporting depression and stress. Overall, diminished research opportunities due to the pandemic have greatly and disproportionately impacted the scientific community, with a higher toll on female scientists (34).

### Residency seniority

Residency year had different levels of association with mental health issues. It is important to note that the residency length and activities are similar throughout the surveyed countries. Residents in the second year and last year of residency showed a higher likelihood of stress. Seniority was considered a risk factor for mental health issues also in a study developed in California on surgical residents. That stated that senior residents tended to work more shifts and their generalized anxiety scores were significantly higher (35).

For second year residents, the pandemic might come as an additional stress factor as in many countries, such as Italy and Spain, the first year is more academic-based (13), which means that the second year starts in a new workplace in a full working hours schedule, being an adaptative period to new tasks and routines, with the COVID-19 pandemic management on top of that.

Within the PHR population, this outcome may occur because of several reasons, one of which being due to more responsibilities given to the last year residents during the COVID pandemic. Also, residents in the last years of their residency are more likely to be looking for a job during this career transition period. During the COVID-19 pandemic, job seeking posed as a great challenge, which can be an additional anxiety risk factor (36).

### Gender gap in mental health outcomes

In the present study, the female gender was associated with negative psychometric outcomes in all the three constructs we investigated: depression, anxiety, and stress. This result is consistent with an abundance of literature of higher prevalence of mental disorders such as depression, anxiety and stress in females compared to males (37, 38). Similar results have been reported in studies that analyzed these factors during the pandemic, showing increased levels of anxiety and depression, with a higher impact in the female group (39). A global study conducted online recurring to DASS-21, concluded that the prevalence of anxiety, stress, and depression was higher in younger people (18–24 years old), female and single, while the presence of family decreased these levels (40).

This result is also supported by many studies conducted on physicians during the COVID-19 pandemic around the world: female doctors were more likely to suffer from depression, anxiety, stress and burnout, and they were associated with worse psychiatric outcomes (6–10, 41). One possible determinant of this gender difference could be that the pandemic has increased not only housework, but also family responsibilities, including childcare needs primarily conducted by women in response to school closures (42). Also, gender discrimination in the workplace can play a critical role on women mental health outcomes. Interestingly, words and actions can negatively impact a woman's wellbeing and success in a way that is often unrecognized outside the experience of a woman herself, and commonly left to the woman to decide how and whether to address it (43). Nevertheless, the risk of response and measurement bias should be considered, since men are less likely to report symptoms and the data were extracted from a self-administered survey (38). It is necessary to further invest in researching the psychological, cultural, and social determinants of this gender difference in terms of mental health outcomes.

### Mental health outcomes: Depression, anxiety, and stress

Overall, the sample featured mild-to-moderate levels of depression and anxiety, and moderate levels of psychological stress. These results are in line with other literature findings, including systematic reviews analyzing Depression, Anxiety, and Stress among healthcare workers during the COVID-19 pandemic. A systematic review analyzed 55 studies across 21 countries involving 97,333 health care workers around the world and overall found a level of moderate depression (21.7%) similar to the prevalence found in the present study (21.9%). The meta-analysis reported a moderate level of anxiety of 22.1 and 27% when only the studies using the DASS-21 questionnaire were included (44). The questionnaire was also applied to Italian health workers, which found that the overall prevalence of moderate-to-extremely severe depression, anxiety, and stress among the 218 participants was 8, 9.8, and 8.9%, respectively: these values are at least threefold lower than the ones we detected in the present study (44.1, 30.1, and 45.4%, respectively). This difference is reduced when focusing on the sample of healthcare workers assisting patients with COVID-19, which reported a prevalence of moderate-to-extremely severe scores ranging from 21.5% for anxiety to 33.4% for stress (45). This difference in findings suggests that the population of doctors in training is experiencing poorer mental health outcomes than the general healthcare worker population.

When comparing our findings to another European study including doctors, nurses, and non-medical professionals answering the DASS-21 questionnaire, results were similar for normal-mild (65%) and moderate (18%) depression, slightly lower for all anxiety levels (68% for normal/mild, 15% for moderate, and 22% for severe anxiety), and comparable for all stress levels (59% for normal/mild, 14% for moderate, and 27% for severe stress) (46).

## Limitations

One of the main limitations of this work is selection bias, as the individuals who agreed to participate in the study may have different characteristics than those who did not. Recall bias should also be mentioned as a limitation, as the Public Health Residents were responding to questions regarding their past experiences. This bias is minimized by the fact that we only inquired about events happening in the previous year. Moreover, the risk of misinterpretation of psychological outcomes must be considered whenever cut-offs are used to define psychological categories, considering the possible overestimation or underestimation of the individual's psychological status. The convenience sampling method does not guarantee the representativeness of the population, and there might be a lower external validity when extrapolating the results. Nevertheless, the big sample size ensures a good generalization of results. The cross-sectional design represents a limitation, impairing causal inference, but the robust statistical analysis allows for the identification of associated factors and determinants of mental health. In the end, a possible limitation may be due to the lack of comparative analysis between the countries that, even if all part of the European Union, may have had different factors impacting residents' mental health, in consideration of different level of preparedness to the pandemic and different approaches of response to it, that were not investigated in this study.

### Strengths

This study has several strengths. First, to our knowledge, this is the first study to assess the initial impact of the COVID-19 pandemic in the Medical Residents in Public Health in Europe.

The participants were from several European countries (France, Italy, Portugal, and Spain). These countries share social and cultural similarities, and all have a National Health Service, as well as similar public health training within the medical residency. We have a large sample size allowing for in-depth statistical analysis. The statistical methods we used to identify associations are robust and correspond well to the study's objectives.

## Conclusion

The COVID-19 pandemic has had a major impact on PHR's mental health and training. This study defines the levels of depression, anxiety, and stress among public health residents, with the main risk factors associated with negative psychometric outcomes being female gender, training challenges, loss of research opportunities, and residency seniority.

It is crucial for PHR to have access not only to Public Health education, but also to counseling and mental health support when needed. Concrete efforts should be deployed into building healthy work environments, especially for women residents, and propose more training and research opportunities.

Moreover, it is essential to increase knowledge and awareness about the impact of this kind of global health emergency on the mental wellbeing of the future Public Health workforce, focusing on vulnerable groups as identified by this research.

## Data availability statement

The original contributions presented in the study are included in the article/Supplementary material, further inquiries can be directed to the corresponding author.

## Author contributions

All authors contributed to revise work for important intellectual content, gave the final approval of the version to be published, and agreed on all aspects of the work, especially concerning its accuracy and integrity.
