# Supplementary material for: Impact of the COVID pandemic on mental health and training opportunities of Public Health Residents from 4 European countries: A cross-sectional study
Source: Front Public Health. 2023 Mar 7;11:1044171. doi: 10.3389/fpubh.2023.1044171 (PMC10028077; doi:10.3389/fpubh.2023.1044171)
Supplement: Supplementary file 1 [file Data_Sheet_1.pdf]

Depression Anxiety Stress Scale Short Version (DASS-21) questionnaire in its French, Italian, Spanish and Portuguese versions (Public Health Residents’ Mental Health, Europe, 2021-2022)

Questionnaire - Santé mentale chez les internes de santé publique en Europe.

Le but de ce questionnaire est d'évaluer la santé mentale, les mécanismes d'adaptationainsi que la perte ou le gain d'opportunités professionnelles dans la population des internes Européen de santé publique.

Le questionnaire est ciblé sur la période allant de mars 2020 à la date actuelle, veuillezchoisir la réponse qui s'applique le plus à votre cas durant cette période.

Toutes les données que vous saisissez sont totalement anonymes et aucune question nepeut être utilisée pour vous identifier.
Le temps de réponse prévu est de 4 à 6 minutes.

* Champs requis

Clause de non-responsabilité : les données personnelles que nous recueillons par le biais de ce formulaire sont collectées uniquement à des fins de recherche médicale, y compris leurs analyses et leurs publications éventuelle. En fournissant vos données personnelles par le biaisde ce formulaire, vous consentez à ce que vos données soient collectées aux fins susmentionnées par le groupe de chercheurs du projet EuroNET MPRH "COVID-19 related mental health, lost opportunities and coping mechanisms of Public Health Residents in Europe".

L'utilisation des informations fournies dans le formulaire sera conforme à l'objectif du formulaire et ne sera pas utilisée autrement. Toutes les données que vous saisissez sont totalement anonymes et aucune question ne peut être utilisée pour vous identifier. Les données ne seront accessibles qu'aux chercheurs participant à cette enquête. Si vous souhaitez en savoir plus sur la manière dont nous gérons vos données, veuillez nous envoyerun courriel à l'adresse research@euronetmrph.org

1. Donnez-vous votre accord pour l'utilisation des données aux fins indiquées ? *

Marque un seul ovale.

- Oui
- Non

Information Générales

2. 1. Quelle est votre année d'internat ? *

Marque un seul ovale.

- 1
- 2
- 3
- 4
- 5
- 6

3. 2. Quel est votre âge ? (chiffre uniquement) *

____

4. 3. Quel est votre genre ? *

Marque un seul ovale.

- Femme
- Homme
- Autre
- Je ne veux pas le mentionner

5. 4. Vivez-vous :

Marque un seul ovale.

- Seul(e)
- Avec de la famille et/ou compagne/compagnonAvec
- de/des ami(e)(s)
- Je ne veux pas le mentionner

6. 5. Quel est votre état civil ?

Marque un seul ovale.

- Célibataire
- Dans une relation
- Marié(e)
- Divorcé(e)
- Veuf/Veuve
- Je ne veux pas le mentionner

7. 6. Durant cette période (pandémie), vous avez travaillé :

Sélectionner tous les éléments applicables.

- [ ] Au contact direct avec des cas suspects/confirmés de COVID-19
- [ ] A des Tâches liées à COVID-19 sans contact direct avec les patients (par exemple, recherche de contacts, surveillance, tâches organisationnelles, etc.)
- [ ] Autre (veuillez décrire en question 6.1)

8. 6.1 Si "autre" en question 6.

____

9. 7. Travaillez vous actuellement sur un projet lié à la COVID19 ?

Marque un seul ovale.

- Oui
- Non

10. 8. Avez vous eu plus d'opportunités de recherches sous l'effet de la pandémieactuelle ?

Marque un seul ovale.

- Oui
- Non

11. 9. Avez vous eu moins d'opportunités de recherches sous l'effet de la pandémieactuelle ?

Marque un seul ovale.

- Oui
- Non

12. 10. Votre internat de santé publique a-t-il été impacté par la pandémie ?

Marque un seul ovale.

- Oui
- Non

13. 10.1 Si oui, expliquez brièvement comment cela a été impacté

____

Santé mentale, questionnaire EDAS 21	0 : ne s'applique pas du tout à moi<br>1 : s'applique un peu à moi, ou une partie du temps<br>2 : s'applique beaucoup à moi, ou une bonne partie du temps<br>3 : s'applique entièrement à moi, ou la grande majorité du temps

14. 11. J'ai trouvé difficile de décompresser. *

0: ne s'applique pas du tout à moi 1: s'applique un peu à moi, ou une partie du temps2: s'applique beaucoup à moi, ou une bonne partie du temps 3: s'applique entièrement à moi, ou la grande majorité du temps

Marque un seul ovale.

- 0
- 1
- 2
- 3

15. 12. J'ai été conscient(e) d'avoir la bouche sèche. *

0: ne s'applique pas du tout à moi 1: s'applique un peu à moi, ou une partie du temps2: s'applique beaucoup à moi, ou une bonne partie du temps 3: s'applique entièrement à moi, ou la grande majorité du temps

Marque un seul ovale.

- 0
- 1
- 2
- 3

16. 13. J'ai eu l'impression de ne pas pouvoir ressentir d'émotion positive. *

0: ne s'applique pas du tout à moi 1: s'applique un peu à moi, ou une partie du temps2: s'applique beaucoup à moi, ou une bonne partie du temps 3: s'applique entièrement à moi, ou la grande majorité du temps

Marque un seul ovale.

- 0
- 1
- 2
- 3

17. 14. J'ai eu de la difficulté à respirer (par exemple, respirations excessivement rapides, essoufflement sans effort physique). *

0: ne s'applique pas du tout à moi 1: s'applique un peu à moi, ou une partie du temps2: s'applique beaucoup à moi, ou une bonne partie du temps 3: s'applique entièrement à moi, ou la grande majorité du temps

Marque un seul ovale.

- 0
- 1
- 2
- 3

18. 15. J'ai eu de la difficulté à initier de nouvelles activités. *

0: ne s'applique pas du tout à moi 1: s'applique un peu à moi, ou une partie du temps2: s'applique beaucoup à moi, ou une bonne partie du temps 3: s'applique entièrement à moi, ou la grande majorité du temps

Marque un seul ovale.

- 0
- 1
- 2
- 3

19. 16. J'ai eu tendance à réagir de façon exagérée. *

0: ne s'applique pas du tout à moi 1: s'applique un peu à moi, ou une partie du temps2: s'applique beaucoup à moi, ou une bonne partie du temps 3: s'applique entièrement à moi, ou la grande majorité du temps

Marque un seul ovale.

- 0
- 1
- 2
- 3

20. 17. J'ai eu des tremblements (par exemple, des mains). *

0: ne s'applique pas du tout à moi 1: s'applique un peu à moi, ou une partie du temps2: s'applique beaucoup à moi, ou une bonne partie du temps 3: s'applique entièrement à moi, ou la grande majorité du temps

Marque un seul ovale.

- 0
- 1
- 2
- 3

21. 18. J'ai eu l'impression de dépenser beaucoup d'énergie nerveuse. *

0: ne s'applique pas du tout à moi 1: s'applique un peu à moi, ou une partie du temps2: s'applique beaucoup à moi, ou une bonne partie du temps 3: s'applique entièrement à moi, ou la grande majorité du temps

Marque un seul ovale.

- 0
- 1
- 2
- 3

22. 19. Je me suis inquiété(e) en pensant à des situations où je pourrais paniquer et faire de moi un(e) idiot(e). *

0: ne s'applique pas du tout à moi 1: s'applique un peu à moi, ou une partie du temps2: s'applique beaucoup à moi, ou une bonne partie du temps 3: s'applique entièrement à moi, ou la grande majorité du temps

Marque un seul ovale.

- 0
- 1
- 2
- 3

23. 20. J'ai eu le sentiment de ne rien envisager avec plaisir. *

0: ne s'applique pas du tout à moi 1: s'applique un peu à moi, ou une partie du temps 2: s'applique beaucoup à moi, ou une bonne partie du temps 3: s'applique entièrement à moi, ou la grande majorité du temps

Marque un seul ovale.

- 0
- 1
- 2
- 3

24. 21. Je me suis aperçu(e) que je devenais agité(e) *

0: ne s'applique pas du tout à moi 1: s'applique un peu à moi, ou une partie du temps2: s'applique beaucoup à moi, ou une bonne partie du temps 3: s'applique entièrement à moi, ou la grande majorité du temps

Marque un seul ovale.

- 0
- 1
- 2
- 3

25. 22. J'ai eu de la difficulté à me détendre. *

0: ne s'applique pas du tout à moi 1: s'applique un peu à moi, ou une partie du temps2: s'applique beaucoup à moi, ou une bonne partie du temps 3: s'applique entièrement à moi, ou la grande majorité du temps

Marque un seul ovale.

- 0
- 1
- 2
- 3

26. 23. Je me suis senti(e) abattu(e) et triste. *

0: ne s'applique pas du tout à moi 1: s'applique un peu à moi, ou une partie du temps 2: s'applique beaucoup à moi, ou une bonne partie du temps 3: s'applique entièrement à moi, ou la grande majorité du temps

Marque un seul ovale.

- 0
- 1
- 2
- 3

27. 24. J'ai été intolérant(e) à tout ce qui m'empêchait de faire ce que j'avais à faire. *

0: ne s'applique pas du tout à moi 1: s'applique un peu à moi, ou une partie du temps2: s'applique beaucoup à moi, ou une bonne partie du temps 3: s'applique entièrement à moi, ou la grande majorité du temps

Marque un seul ovale.

- 0
- 1
- 2
- 3

28. 25. J'ai eu le sentiment d'être presque pris(e) de panique. *

0: ne s'applique pas du tout à moi 1: s'applique un peu à moi, ou une partie du temps2: s'applique beaucoup à moi, ou une bonne partie du temps 3: s'applique entièrement à moi, ou la grande majorité du temps

Marque un seul ovale.

- 0
- 1
- 2
- 3

29. 26. J'ai été incapable de me sentir enthousiaste au sujet de quoi que ce soit. *

0: ne s'applique pas du tout à moi 1: s'applique un peu à moi, ou une partie du temps 2: s'applique beaucoup à moi, ou une bonne partie du temps 3: s'applique entièrement à moi, ou la grande majorité du temps

Marque un seul ovale.

- 0
- 1
- 2
- 3

30. 27. J'ai eu le sentiment de ne pas valoir grand chose comme personne. *

0: ne s'applique pas du tout à moi 1: s'applique un peu à moi, ou une partie du temps2: s'applique beaucoup à moi, ou une bonne partie du temps 3: s'applique entièrement à moi, ou la grande majorité du temps

Marque un seul ovale.

- 0
- 1
- 2
- 3

31. 28. J'ai eu l'impression d'être assez susceptible. *

0: ne s'applique pas du tout à moi 1: s'applique un peu à moi, ou une partie du temps2: s'applique beaucoup à moi, ou une bonne partie du temps 3: s'applique entièrement à moi, ou la grande majorité du temps

Marque un seul ovale.

- 0
- 1
- 2
- 3

32. 29. J'ai été conscient(e) des palpitations de mon cœur en l'absence d'effort physique (sensation d'augmentation de mon rythme cardiaque ou l'impressionque mon cœur venait de sauter). *

0: ne s'applique pas du tout à moi 1: s'applique un peu à moi, ou une partie du temps2: s'applique beaucoup à moi, ou une bonne partie du temps 3: s'applique entièrement à moi, ou la grande majorité du temps

Marque un seul ovale.

- 0
- 1
- 2
- 3

33. 30. J'ai eu peur sans bonne raison. *

0: ne s'applique pas du tout à moi 1: s'applique un peu à moi, ou une partie du temps2: s'applique beaucoup à moi, ou une bonne partie du temps 3: s'applique entièrement à moi, ou la grande majorité du temps

Marque un seul ovale.

- 0
- 1
- 2
- 3

34. 31. J'ai eu l'impression que la vie n'avait pas de sens. *

0: ne s'applique pas du tout à moi 1: s'applique un peu à moi, ou une partie du temps2: s'applique beaucoup à moi, ou une bonne partie du temps 3: s'applique entièrement à moi, ou la grande majorité du temps

Marque un seul ovale.

- 0
- 1
- 2
- 3

35. 32. Si vous le souhaitez, vous pouvez partager toute information brève et pertinente relative à votre santé mentale, mécanismes d'adaptation ou opportunités professionnelles pendant la crise lié a la COVID-19.

Salute Mentale degli/delle Specializzandi/e in Sanità Pubblica

Lo scopo di questo questionario è valutare la salute mentale, e le opportunità perse,oppure ottenute, tra gli/le specializzandi/e in Sanità Pubblica in Europa, durante la pandemia da SARS-CoV2.
Il questionario si riferisce al periodo che va da Marzo 2020 ad oggi, si prega di risponderein modo più accurato possibile riguardo alla vostra situazione durante tale periodo.
Tutti i dati che fornirete rientrano completamente nell'anonimato rispettando la vostraprivacy e nessuna domanda e/o risposta verrà utilizzata per identificarvi.
Il questionario richiede in media 4-6 minuti per essere compilato.

*Campo obbligatorio

Disclaimer

I dati identificabili di questo questionario saranno archiviati esclusivamente per scopi diricerca, incluse le analisi e la potenziale pubblicazione di un articolo collegato.
Fornendo i tuoi dati con questo form acconsenti che i tuoi dati siano utilizzati per gli scopicitati dai ricercatori di EuroNET MPRH Project impegnati nel progetto: "COVID-19 relatedmental health, lost opportunities and coping mechanisms of Public Health Residents in Europe".
L'uso delle informazioni fornite sarà in linea con lo scopo dello studio e non potrà essere usatoin alcun modo al di fuori di questo.
Tutti i dati forniti saranno completamente anonimi e nessuna risposta potrà essere collegata ate.

I dati saranno archiviati fino a Dicembre 2025 e saranno accessibili solo dai creatori di questoform, che sono anche i ricercatori del gruppo prima citato.
Se sei interessato a conoscere nel dettaglio come verranno utilizzati i dati, sentiti libero discriverci a: research@euronetmrph.org

1. Presti il tuo consenso per gli usi dei dati indicati nel disclaimer? *

Contrassegna solo un ovale.

- ◯ Sì
- ◯ No

Informazioni Generali

2. 1. A quale anno di Specializzazione sei iscritto/a? *

Contrassegna solo un ovale.

- 1
- 2
- 3
- 4

3. 2. Quanti anni hai? (in numero) *

____

4. 3. Con quale genere ti identifichi? *

Contrassegna solo un ovale.

- Femminile
- Maschile
- Altro
- Preferisco non specificare

5. 4. Con chi vivi? *

1= Da Sol* ; 2: Con la mia Famiglia e/o partner ; 3: Con Amici ; 4: Preferisco non indicarlo

Contrassegna solo un ovale.

- 1
- 2
- 3
- 4

6. 5. Quale è la tua situazione sentimentale? *

1: Single ; 2: In una Relazione; 3: Sposat* ; 4: Divorziat*; 5: Vedov* ; 6: Preferisco non indicarlo

Contrassegna solo un ovale.

- 1
- 2
- 3
- 4
- 5
- 6

7. 6. Dall'inizio della Pandemia di COVID-19, hai mai lavorato in prima linea (in contatto diretto di certo o presunto caso di paziente affetto da COVID-19)? *

Contrassegna solo un ovale.

- Contatto diretto con caso accertato/sospetto di COVID-19
- In attività correlate al COVID-19 senza contatto diretto con i pazienti (ad esempio tracciamento dei contatti, sorveglianza, attività organizzative, ecc.)
- Altro: ____________________

8. 7. Hai lavorato per un progetto riguardo al COVID-19? *

0=NO ; 1=SÌ

Contrassegna solo un ovale.

- 0
- 1

9. 8. Hai avuto più opportunità di fare ricerca dopo l'inizio della pandemia da COVID-19? *

0=NO ; 1=SÌ

Contrassegna solo un ovale.

- 0
- 1

10. 9. Hai avuto meno opportunità di fare ricerca dopo l'inizio della pandemia da COVID-19? *

0=NO ; 1=SÌ

Contrassegna solo un ovale.

- 0
- 1

11. 10. La tua formazione da specializzando in sanità pubblica ha risentito della pandemia da COVID-19? *

0=NO ; 1=SÌ

Contrassegna solo un ovale.

- 0
- 1

12. 10.1 Se hai risposto "1=sì" descrivi brevemente come è stata influenzata la tua formazione

____

____

____

____

____

Salute Mentale I

Questa parte si concentra sull'assessment della salute mentalesecondo la scala DASS-21

13. 11. Ho avuto difficoltà ad allentare la tensione *

(0=Mai ; 1=Qualche volta ; 2=Spesso ; 3=Quasi sempre)

Contrassegna solo un ovale.

	0	1	2	3
	⬭	⬭	⬭	⬭

14. 12. Mi sono accorto/a di avere la bocca secca *

(0=Mai ; 1=Qualche volta ; 2=Spesso ; 3=Quasi sempre)

Contrassegna solo un ovale.

	0	1	2	3
	⬭	⬭	⬭	⬭

15. 13. Non riuscivo proprio a provare delle emozioni positive *

(0=Mai ; 1=Qualche volta ; 2=Spesso ; 3=Quasi sempre)

Contrassegna solo un ovale.

	0	1	2	3
	⬭	⬭	⬭	⬭

16. 14. Ho avuto difficoltà nella respirazione (per es. respiro molto accelerato, sensazione di forte affanno in assenza di sforzo fisico) *

(0=Mai ; 1=Qualche volta ; 2=Spesso ;3=Quasi sempre)

Contrassegna solo un ovale.

0	1	2	3

17. 15. Ho avuto difficoltà nel cominciare quello che dovevo fare *

(0=Mai ; 1=Qualche volta ; 2=Spesso ; 3=Quasi sempre)

Contrassegna solo un ovale.

0	1	2	3

18. 16. Ho avuto la tendenza a reagire in maniera eccessiva alle situazioni *

(0=Mai ; 1=Qualche volta ; 2=Spesso ; 3=Quasi sempre)

Contrassegna solo un ovale.

0	1	2	3

19. 17. Ho avuto tremori (per es. alle mani) *

(0=Mai ; 1=Qualche volta ; 2=Spesso ; 3=Quasi sempre)

Contrassegna solo un ovale.

0	1	2	3

20. 18. Ho sentito che stavo impiegando molta energia nervosa *

(0=Mai ; 1=Qualche volta ; 2=Spesso ; 3=Quasi sempre)

Contrassegna solo un ovale.

0	1	2	3
⬭	⬭	⬭	⬭

21. 19. Ho temuto di trovarmi in situazioni in cui sarei potuto andare nel panico e rendermi ridicolo *

(0=Mai ; 1=Qualche volta ; 2=Spesso ; 3=Quasi sempre)

Contrassegna solo un ovale.

0	1	2	3
⬭	⬭	⬭	⬭

22. 20. Non vedevo nulla di buono nel mio futuro *

(0=Mai ; 1=Qualche volta ; 2=Spesso ; 3=Quasi sempre)

Contrassegna solo un ovale.

0	1	2	3
⬭	⬭	⬭	⬭

23. 21. Mi sono sentito stressato/a *

(0=Mai ; 1=Qualche volta ; 2=Spesso ; 3=Quasi sempre)

Contrassegna solo un ovale.

0	1	2	3
⬭	⬭	⬭	⬭

24. 22. Ho avuto difficoltà a rilassarmi *
(0=Mai ; 1=Qualche volta ; 2=Spesso ; 3=Quasi sempre)

Contrassegna solo un ovale.

0	1	2	3
⬭	⬭	⬭	⬭

25. 23. Mi sono sentito scoraggiato/a e depresso/a *
(0=Mai ; 1=Qualche volta ; 2=Spesso ; 3=Quasi sempre)

Contrassegna solo un ovale.

0	1	2	3
⬭	⬭	⬭	⬭

26. 24. Non riuscivo a tollerare per nulla eventi o situazioni che mi impedivano di portare avanti ciò che stavo facendo *
(0=Mai ; 1=Qualche volta ; 2=Spesso ; 3=Quasi sempre)

Contrassegna solo un ovale.

0	1	2	3
⬭	⬭	⬭	⬭

27. 25. Ho sentito di essere vicino ad avere un attacco di panico *
(0=Mai ; 1=Qualche volta ; 2=Spesso ; 3=Quasi sempre)

Contrassegna solo un ovale.

0	1	2	3
⬭	⬭	⬭	⬭

28. 26. Non c’era nulla che mi dava entusiasmo *

(0=Mai ; 1=Qualche volta ; 2=Spesso ; 3=Quasi sempre)

Contrassegna solo un ovale.

0	1	2	3
◯	◯	◯	◯

29. 27. Sentivo di valere poco come persona *

(0=Mai ; 1=Qualche volta ; 2=Spesso ; 3=Quasi sempre)

Contrassegna solo un ovale.

0	1	2	3
◯	◯	◯	◯

30. 28. Mi sono sentito piuttosto irritabile *

(0=Mai ; 1=Qualche volta ; 2=Spesso ; 3=Quasi sempre)

Contrassegna solo un ovale.

0	1	2	3
◯	◯	◯	◯

31. 29. Ho percepito distintamente il battito del mio cuore senza aver fatto uno sforzo fisico (per es. battito cardiaco accelerato o perdita di un battito) *

(0=Mai ; 1=Qualche volta ; 2=Spesso ; 3=Quasi sempre)

Contrassegna solo un ovale.

0	1	2	3
◯	◯	◯	◯

32. 30. Mi sono sentito spaventato/a senza ragione *

(0=Mai ; 1=Qualche volta ; 2=Spesso ; 3=Quasi sempre)

Contrassegna solo un ovale.

0	1	2	3
⬭	⬭	⬭	⬭

33. 31. Sentivo la vita priva di significato *

(0=Mai ; 1=Qualche volta ; 2=Spesso ; 3=Quasi sempre)

Contrassegna solo un ovale.

0	1	2	3
⬭	⬭	⬭	⬭

Inquérito – Saúde Mental em Médicos Internos de Saúde Pública durante a pandemia da COVID-19

O objetivo deste questionário é avaliar a Saúde Mental, e a perda ou o ganho de oportunidades formativas devido à pandemia de COVID-19, na população de Médicos Internos de Saúde Pública em Portugal, Espanha, França e Itália.
O questionário diz respeito ao período de março de 2020 até ao momento atual. Por favor, escolhe a resposta que mais se aplica ao teu caso durante este período.
Todos os dados inseridos são completamente anónimos e nenhuma pergunta pode ser usada para te identificar.
O tempo de preenchimento do inquérito é de cerca de 5 minutos.

---

*Campo obbligatorio

Nota: Os dados pessoais que colhemos através deste formulário são colhidos apenas para fins de investigação médica, incluindo a sua análise e possível publicação. Ao fornecer os seus dados pessoais através deste formulário, estás a dar o teu consentimento para que os teus dados sejam recolhidos para os fins anteriormente declarados para os investigadores do Projecto EuroNET MPRH "COVID-19 relacionado com a saúde mental, oportunidades perdidas e mecanismos de coping em médicos internos de saúde pública na Europa". A utilização das informações fornecidas no formulário está em conformidade com a finalidade do formulário e não será utilizada de outra forma. Todos os dados inseridos são completamente anónimos e nenhuma pergunta pode ser usada para identificar-te. Os dados serão armazenados até dezembro de 2025 e só poderão ser acedidos pelos criadores e investigadores deste estudo. Se quiser saber mais sobre como gerimos os teus dados, envie um e-mail para research@euronetmrph.org

1. Concordas com o uso de dados para os fins indicados? *

Contrassegna solo un ovale.

- Sim
- Não

Informação geral

2. 1. Em que ano do internato te encontras? *

Contrassegna solo un ovale.

- 1
- 2
- 3
- 4

3. 2. Qual é a tua idade? *

____

4. 3. Qual é o teu género? *

Contrassegna solo un ovale.

- Feminino
- Masculino
- Outro
- Prefiro não dizer

5. 4. Com quem vives? *

Contrassegna solo un ovale.

- Sozinho
- Com família e/ou parceiro(a)
- Com amigos
- Prefiro não dizer

6. 5. Qual é o teu estado civil? *

Contrassegna solo un ovale.

- Solteiro(a)
- Num relacionamento
- Casado(a) ou em união de facto
- Divorciado(a)
- Viúvo(a)
- Prefiro não dizer

7. 6. Desde o início da pandemia por COVID-19, tens trabalhado em: *

Seleziona tutte le voci applicabili.

- Contacto direto com pessoas com COVID-19 (casos confirmados ou suspeitos)
- Tarefas relacionadas com o COVID-19 que não envolvem contacto direto com doentes (ex: rastreio de contactos, vigilância epidemiológica, tarefas organizacionais, etc)
- Altro: ______________________

8. 7. Estás a trabalhar em algum projeto relacionado com a COVID-19?

Contrassegna solo un ovale.

- Sim
- Não

9. 8. Tiveste mais oportunidades de investigação devido à pandemia COVID-19? *

Contrassegna solo un ovale.

- Sim
- Não

10. 9. Tiveste menos oportunidades de investigação devido à pandemia por COVID-19? *

Contrassegna solo un ovale.

- Sim
- Não

11. 10. A tua formação em Saúde Pública foi afetada pela pandemia por COVID-19?

Contrassegna solo un ovale.

- Sim
- Não *Passa alla domanda 13.*

12. 10.1 Em caso afirmativo, explica brevemente como foi afetada.

____

Saúde Mental (Escala DASS-21)

0 : Nunca

1: Às vezes

2: Frequentemente

3: Quase sempre

13. 11. Tive dificuldades em acalmar-me. *

0: Nunca 1: Às vezes. 2: Frequentemente 3: Quase sempre

Contrassegna solo un ovale.

- 0
- 1
- 2
- 3

14. 12. Senti a boca seca. *

0: Nunca 1: Às vezes. 2: Frequentemente 3: Quase sempre

Contrassegna solo un ovale.

- 0
- 1
- 2
- 3

15. 13. Não consegui sentir nenhum sentimento positivo. *

0: Nunca 1: Às vezes. 2: Frequentemente 3: Quase sempre

Contrassegna solo un ovale.

- 0
- 1
- 2
- 3

16. 14. Senti dificuldades em respirar. *

0: Nunca 1: Às vezes. 2: Frequentemente 3: Quase sempre

Contrassegna solo un ovale.

- 0
- 1
- 2
- 3

17. 15. Tive dificuldade em tomar iniciativa para fazer coisas. *

0: Nunca 1: Às vezes. 2: Frequentemente 3: Quase sempre

Contrassegna solo un ovale.

- 0
- 1
- 2
- 3

18. 16. Tive tendência a reagir em demasia em determinadas situações. *

0: Nunca 1: Às vezes. 2: Frequentemente 3: Quase sempre

Contrassegna solo un ovale.

- 0
- 1
- 2
- 3

19. 17. Senti tremores (por ex., nas mãos). *

0: Nunca 1: Às vezes. 2: Frequentemente 3: Quase sempre

Contrassegna solo un ovale.

- 0
- 1
- 2
- 3

20. 18. Senti que estava sempre nervoso. *

0: Nunca 1: Às vezes. 2: Frequentemente 3: Quase sempre

Contrassegna solo un ovale.

- 0
- 1
- 2
- 3

21. 19. Preocupei-me com situações em que podia entrar em pânico e fazer figura ridícula. *

0: Nunca 1: Às vezes. 2: Frequentemente 3: Quase sempre

Contrassegna solo un ovale.

- 0
- 1
- 2
- 3

22. 20. Senti que não tinha nada a esperar do futuro. *

0: Nunca 1: Às vezes. 2: Frequentemente 3: Quase sempre

Contrassegna solo un ovale.

- 0
- 1
- 2
- 3

23. 21. Dei por mim a ficar agitado. *

0: Nunca 1: Às vezes. 2: Frequentemente 3: Quase sempre

Contrassegna solo un ovale.

- 0
- 1
- 2
- 3

24. 22. Senti dificuldade em relaxar. *

0: Nunca 1: Às vezes. 2: Frequentemente 3: Quase sempre

Contrassegna solo un ovale.

- 0
- 1
- 2
- 3

25. 23. Senti-me desanimado e melancólico. *

0: Nunca 1: Às vezes. 2: Frequentemente 3: Quase sempre

Contrassegna solo un ovale.

- 0
- 1
- 2
- 3

26. 24. Fui intolerante em relação a qualquer coisa que me impedisse de terminar aquilo que estava a fazer. *

0: Nunca 1: Às vezes. 2: Frequentemente 3: Quase sempre

Contrassegna solo un ovale.

- 0
- 1
- 2
- 3

27. 25. Senti-me quase a entrar em pânico. *

0: Nunca 1: Às vezes. 2: Frequentemente 3: Quase sempre

Contrassegna solo un ovale.

- 0
- 1
- 2
- 3

28. 26. Não fui capaz de ter entusiasmo por nada. *

0: Nunca 1: Às vezes. 2: Frequentemente 3: Quase sempre

Contrassegna solo un ovale.

- 0
- 1
- 2
- 3

29. 27. Senti que não tinha muito valor como pessoa. *

0: Nunca 1: Às vezes. 2: Frequentemente 3: Quase sempre

Contrassegna solo un ovale.

- 0
- 1
- 2
- 3

30. 28. Senti que por vezes estava sensível. *

0: Nunca 1: Às vezes. 2: Frequentemente 3: Quase sempre

Contrassegna solo un ovale.

- 0
- 1
- 2
- 3

31. 29. Senti alterações no meu coração mesmo não tendo feito exercício físico. (ex. aumento da frequência cardíaca, disritmia cardíaca) *

0: Nunca 1: Às vezes. 2: Frequentemente 3: Quase sempre

Contrassegna solo un ovale.

- 0
- 1
- 2
- 3

32. 30. Senti-me assustado sem ter tido uma boa razão para isso. *

0: Nunca 1: Às vezes. 2: Frequentemente 3: Quase sempre

Contrassegna solo un ovale.

- 0
- 1
- 2
- 3

33. 31. Senti que a vida não tinha sentido. *

0: Nunca 1: Às vezes. 2: Frequentemente 3: Quase sempre

Contrassegna solo un ovale.

- 0
- 1
- 2
- 3

34. 32. Se quiseres, podes partilhar alguma informação curta ou relevante sobre a tua Saúde Mental, mecanismos de coping e oportunidades durante a pandemia por COVID-19.

Questi contenuti non sono creati né avallati da Google.

Google Moduli

Cuestionario de evaluación de la salud mental de los y las residentes de Medicina Preventiva y Salud Pública (España)

El propósito de este cuestionario es evaluar la salud mental, los mecanismos de afrontamiento y la pérdida o ganancia de oportunidades entre los médicos residentes deMedicina Preventiva y Salud Pública en Portugal, España, Francia e Italia durante la pandemia de COVID-19.

El período a estudio es el comprendido entre marzo de 2020 y el momento actual. Elige larespuesta que más se ajuste a tu situación durante este período.

Todos los datos registrados son completamente anónimos, y no se podrá utilizar ningunapregunta para identificarte.

El tiempo estimado para su realización es entre 4 y 6 minutos.

*Campo requerido

Descargo de responsabilidad - Los datos que se recopilan a través de este formulario únicamente se utilizarán en esta investigación, incluido su análisis y posible publicación de resultados. Al proporcionar sus datos personales a través de este formulario estás dando tu consentimiento para que se utilicen, con los fines anteriormente indicados, a los y las investigadoras del proyecto de EuroNET MPRH: "Salud mental relacionada con la COVID-19, oportunidades perdidas y mecanismos de afrontamiento de los y las médicos residentes de Medicina Preventiva y Salud Pública en Portugal, España, Francia y Italia". El uso de la información proporcionada en el formulario estará en línea con el propósito del mismo y no se utilizará de otra manera. Todos los datos que registres serán completamente anónimos y no se podrá utilizar ninguna pregunta para identificarte. Los datos se almacenarán hasta diciembrede 2025 y solo serán accesibles para los creadores e investigadores de este proyecto.Si deseas saber más sobre cómo administramos tus datos, envíanos un correo electrónico a research@euronetmrph.org.

1. ¿Das tu consentimiento para el uso de tus dados con el objetivo mencionado? *

Marca solo un ovalo.

- Si
- No

Informacion General

2. 1. ¿En qué año de residencia te encuentras? *

Marca solo un ovalo.

- 1
- 2
- 3
- 4
- 5

3. 2. ¿Qué edad tienes? *

____

4. 3. ¿Cual es tu género? *

Marca solo un ovalo.

- Femenino
- Masculino
- Otro
- Prefiero no decirlo

5. 4. ¿Con quién vives habitualmente? *

Marca solo un ovalo.

- Solo(a)
- Con familia y/o compañero(a) sentimentalCon
- amigos(as)
- Prefiero no decirlo

6. 5. ¿Cuál es tu estado civil? *

Marca solo un ovalo.

- Soltero(a)
- En una relación
- Casado(a)
- Divorciado(a)
- Viudo(a)
- Prefiero no decirlo

7. 6. Durante este período has trabajado en: *

Seleccione todos los elementos aplicables

- [ ] Contacto directo con casos sospechosos/confirmados de COVID-19
- [ ] Tareas relacionadas con COVID-19 sin contacto directocon pacientes (por ejemplo, seguimiento de contactos, vigilancia, tareas organizativas, etc)
- [ ] Otras (por favor, descríbelas)

8. 7. ¿Estás trabajando actualmente en un proyecto relacionado con la COVID-19?

Marca solo un ovalo.

- Si
- No

9. 8. ¿Consideras que has tenido más oportunidades de investigación durante este período? *

Marca solo un ovalo.

- Si
- No

10. 9. ¿Consideras que has tenido menos oportunidades de investigación durante este período? *

Marca solo un ovalo.

- Si
- No

11. 10. ¿Consideras que la pandemia ha tenido algún impacto en tu residencia?

Marca solo un ovalo.

- Si
- No *Ir a la pregunta 13*

12. 10.1 Si consideras que ha tenido algún impacto, por favor, descríbelo.

____

Salud Mental (Cuestionario DASS-21)

0 : Nunca

1: Algunas veces

2: Muchas veces

3: Casi siempre

13. 11. Me ha costado mucho descargar la tensión. *

0: Nunca 1: Algunas veces. 2: Muchas veces 3: Casi siempre

Marca solo un ovalo.

- 0
- 1
- 2
- 3

14. 12. Me di cuenta que tenía la boca seca. *

0: Nunca 1: Algunas veces. 2: Muchas veces 3: Casi siempre

Marca solo un ovalo.

- 0
- 1
- 2
- 3

15. 13. No podía sentir ningún sentimiento positivo. *

0: Nunca 1: As veces 2: Muchas vieces 3: Casi siempre

Marca solo un ovalo.

- 0
- 1
- 2
- 3

16. 14. Se me hizo difícil respirar. *

0: Nunca 1: Algunas veces. 2: Muchas veces 3: Casi siempre

Marca solo un ovalo.

- 0
- 1
- 2
- 3

17. 15. Se me hizo difícil tomar la iniciativa para hacer cosas. *

0: Nunca 1: Algunas veces. 2: Muchas veces 3: Casi siempre

Marca solo un ovalo.

- 0
- 1
- 2
- 3

18. 16. Reaccioné exageradamente en ciertas situaciones. *

0: Nunca 1: Algunas veces. 2: Muchas veces 3: Casi siempre

Marca solo un ovalo.

- 0
- 1
- 2
- 3

19. 17. Sentí que mis manos temblaban. *

0: Nunca 1: Algunas veces. 2: Muchas veces 3: Casi siempre

Marca solo un ovalo.

- 0
- 1
- 2
- 3

20. 18. He sentido que estaba gastando una gran cantidad de energía. *

0: Nunca 1: Algunas veces. 2: Muchas veces 3: Casi siempre

Marca solo un ovalo.

- 0
- 1
- 2
- 3

21. 19. Estaba preocupado por situaciones en las cuales podía tener pánico o en las que podríahacer el ridículo. *

0: Nunca 1: Algunas veces. 2: Muchas veces 3: Casi siempre

Marca solo un ovalo.

- 0
- 1
- 2
- 3

22. 20. He sentido que no había nada que me ilusionara. *

0: Nunca 1: Algunas veces. 2: Muchas veces 3: Casi siempre

Marca solo un ovalo.

- 0
- 1
- 2
- 3

23. 21. Me he sentido inquieto. *

0: Nunca 1: Algunas veces. 2: Muchas veces 3: Casi siempre

Marca solo un ovalo.

- 0
- 1
- 2
- 3

24. 22. Se me hizo difícil relajarme. *

0: Nunca 1: Algunas veces. 2: Muchas veces 3: Casi siempre

Marca solo un ovalo.

- 0
- 1
- 2
- 3

25. 23. Me sentí triste y deprimido. *

0: Nunca 1: Algunas veces. 2: Muchas veces 3: Casi siempre

Marca solo un ovalo.

- 0
- 1
- 2
- 3

26. 24. No toleré nada que no me permitiera continuar con lo que estaba haciendo. *

0: Nunca 1: Algunas veces. 2: Muchas veces 3: Casi siempre

Marca solo un ovalo.

- 0
- 1
- 2
- 3

27. 25. Sentí que estaba al punto de pánico. *

0: Nunca 1: Algunas veces. 2: Muchas veces 3: Casi siempre

Marca solo un ovalo.

- 0
- 1
- 2
- 3

28. 26. No me pude entusiasmar por nada. *

0: Nunca 1: Algunas veces. 2: Muchas veces 3: Casi siempre

Marca solo un ovalo.

- 0
- 1
- 2
- 3

29. 27. Sentí que valía muy poco como persona. *

0: Nunca 1: Algunas veces. 2: Muchas veces 3: Casi siempre

Marca solo un ovalo.

- 0
- 1
- 2
- 3

30. 28. He tendido a sentirme enfadado con facilidad. *

0: Nunca 1: Algunas veces. 2: Muchas veces 3: Casi siempre

Marca solo un ovalo.

- 0
- 1
- 2
- 3

31. 29. Sentí los latidos de mi corazón a pesar de no haber hecho ningún esfuerzo * físico.

0: Nunca 1: Algunas veces. 2: Muchas veces 3: Casi siempre

Marca solo un ovalo.

- 0
- 1
- 2
- 3

32. 30. Tuve miedo sin razón. *

0: Nunca 1: Algunas veces. 2: Muchas veces 3: Casi siempre

Marca solo un ovalo.

- 0
- 1
- 2
- 3

33. 31. Sentí que la vida no tenía ningún sentido. *

0: Nunca 1: Algunas veces. 2: Muchas veces 3: Casi siempre

Marca solo un ovalo.

- 0
- 1
- 2
- 3

Información Adicional

34. 32. Si lo deseas, puedes compartir cualquier información breve y relevante relacionada con tu salud mental, mecanismos de afrontamiento u oportunidadesdurante la COVID-19.
