# Supplementary material for: Impact of the COVID pandemic on mental health and training opportunities of Public Health Residents from 4 European countries: A cross-sectional study
Source: Front Public Health. 2023 Mar 7;11:1044171. doi: 10.3389/fpubh.2023.1044171 (PMC10028077; doi:10.3389/fpubh.2023.1044171)
Supplement: Supplementary file 2 [file Data_Sheet_2.pdf]

Questionnaire – Mental Health in Public Health Residents

The purpose of this questionnaire is to assess the mental health, coping mechanisms and loss or gain in opportunities in the population of medical public health residents in Europe.

The questionnaire is targeted at the period from March 2020 to this date, please choose the answer that applies the most to your case during this period.

All data you enter is completely anonymous and no question can be used to identify you.

The expected time of completion is of 8-10 minutes.

General information

1. What is your year of residency?

   ____________ *(continuous variable)*

2. What is your age?

   ____________ *(continuous variable)*

3. What is the gender you identify with?

   ◻ F ◻ M ◻ Other ◻ I'd rather not tell

4. What's your living arrangement?

◻ Living alone ◻ Living with family ◻ Living with friends ◻ Prefer not to tell

5. What is your relationship status?

◻ Single ◻ In a couple ◻ Married ◻ Divorced ◻ Widowed ◻ Prefer not to tell

6. Since the COVID-19 pandemic started, have you been working in the frontline (in direct contact with positive or presumptive COVID-19 cases)?

   ◻ Y ◻ N

7. Have you been working on a COVID-19-related project?

   ◻ Y ◻ N

Opportunities

8. Did you get more research opportunities due to the COVID-19 pandemic?

   ◻ Y ◻ N

9. Did you get less research opportunities due to the COVID-19 pandemic?

   ◻ Y ◻ N

10. Was your Public Health training impacted because of the pandemic?

   ◻ Y ◻ N *(if Y, question 8.1 appears, if N, skip to question 9)*

   10.1. If yes, please explain briefly how it was impacted.

   ____

Mental health (DASS-21 scale)

11. I found it hard to wind down

   ◻ Never ◻ Sometimes ◻ Often ◻ Almost always

12. I was aware of dryness of my mouth
□ Never □ Sometimes □ Often □ Almost always
13. I couldn't seem to experience any positive feeling at all
□ Never □ Sometimes □ Often □ Almost always
14. I experienced breathing difficulty (eg, excessively rapid breathing, breathlessness in the absence of physical exertion)
□ Never □ Sometimes □ Often □ Almost always
15. I found it difficult to work up the initiative to do things
□ Never □ Sometimes □ Often □ Almost always
16. I tended to over-react to situations
□ Never □ Sometimes □ Often □ Almost always
17. I experienced trembling (eg, in the hands)
□ Never □ Sometimes □ Often □ Almost always
18. I felt that I was using a lot of nervous energy
□ Never □ Sometimes □ Often □ Almost always
19. I was worried about situations in which I might panic and make a fool of myself
□ Never □ Sometimes □ Often □ Almost always
20. I felt that I had nothing to look forward to
□ Never □ Sometimes □ Often □ Almost always
21. I found myself getting agitated
□ Never □ Sometimes □ Often □ Almost always
22. I found it difficult to relax
□ Never □ Sometimes □ Often □ Almost always
23. I felt down-hearted and blue
□ Never □ Sometimes □ Often □ Almost always
24. I was intolerant of anything that kept me from getting on with what I was doing
□ Never □ Sometimes □ Often □ Almost always
25. I felt I was close to panic
□ Never □ Sometimes □ Often □ Almost always
26. I was unable to become enthusiastic about anything
□ Never □ Sometimes □ Often □ Almost always
27. I felt I wasn't worth much as a person
□ Never □ Sometimes □ Often □ Almost always
28. I felt that I was rather touchy
□ Never □ Sometimes □ Often □ Almost always
29. I was aware of the action of my heart in the absence of physical exertion (eg, sense of heart rate increase, heart missing a beat)
□ Never □ Sometimes □ Often □ Almost always
30. I felt scared without any good reason
□ Never □ Sometimes □ Often □ Almost always
31. I felt that life was meaningless
□ Never □ Sometimes □ Often □ Almost always

Coping mechanisms (brief COPE)

32. I've been turning to work or other activities to take my mind off things.
    □ I haven't been doing this at all □ I've been doing this a little bit
    □ I've been doing this a medium amount □ I've been doing this a lot
33. I've been concentrating my efforts on doing something about the situation I'm in.
    □ I haven't been doing this at all □ I've been doing this a little bit
    □ I've been doing this a medium amount □ I've been doing this a lot
34. I've been saying to myself "this isn't real".
    □ I haven't been doing this at all □ I've been doing this a little bit
    □ I've been doing this a medium amount □ I've been doing this a lot
35. I've been using alcohol or other drugs to make myself feel better.
    □ I haven't been doing this at all □ I've been doing this a little bit
    □ I've been doing this a medium amount □ I've been doing this a lot
36. I've been getting emotional support from others.
    □ I haven't been doing this at all □ I've been doing this a little bit
    □ I've been doing this a medium amount □ I've been doing this a lot
37. I've been giving up trying to deal with it.
    □ I haven't been doing this at all □ I've been doing this a little bit
    □ I've been doing this a medium amount □ I've been doing this a lot
38. I've been taking action to try to make the situation better.
    □ I haven't been doing this at all □ I've been doing this a little bit
    □ I've been doing this a medium amount □ I've been doing this a lot
39. I've been refusing to believe that it has happened.
    □ I haven't been doing this at all □ I've been doing this a little bit
    □ I've been doing this a medium amount □ I've been doing this a lot
40. I've been saying things to let my unpleasant feelings escape.
    □ I haven't been doing this at all □ I've been doing this a little bit
    □ I've been doing this a medium amount □ I've been doing this a lot
41. I've been getting help and advice from other people.
    □ I haven't been doing this at all □ I've been doing this a little bit
    □ I've been doing this a medium amount □ I've been doing this a lot
42. I've been using alcohol or other drugs to help me get through it.
    □ I haven't been doing this at all □ I've been doing this a little bit
    □ I've been doing this a medium amount □ I've been doing this a lot
43. I've been trying to see it in a different light, to make it seem more positive.
    □ I haven't been doing this at all □ I've been doing this a little bit
    □ I've been doing this a medium amount □ I've been doing this a lot
44. I've been criticizing myself.
    □ I haven't been doing this at all □ I've been doing this a little bit
    □ I've been doing this a medium amount □ I've been doing this a lot

45. I've been trying to come up with a strategy about what to do.
□ I haven't been doing this at all □ I've been doing this a little bit
□ I've been doing this a medium amount □ I've been doing this a lot
46. I've been getting comfort and understanding from someone.
□ I haven't been doing this at all □ I've been doing this a little bit
□ I've been doing this a medium amount □ I've been doing this a lot
47. I've been giving up the attempt to cope.
□ I haven't been doing this at all □ I've been doing this a little bit
□ I've been doing this a medium amount □ I've been doing this a lot
48. I've been looking for something good in what is happening.
□ I haven't been doing this at all □ I've been doing this a little bit
□ I've been doing this a medium amount □ I've been doing this a lot
49. I've been making jokes about it.
□ I haven't been doing this at all □ I've been doing this a little bit
□ I've been doing this a medium amount □ I've been doing this a lot
50. I've been doing something to think about it less, such as watching movies, watching TV, reading, daydreaming, sleeping, or shopping.
□ I haven't been doing this at all □ I've been doing this a little bit
□ I've been doing this a medium amount □ I've been doing this a lot
51. I've been accepting the reality of the fact that it has happened.
□ I haven't been doing this at all □ I've been doing this a little bit
□ I've been doing this a medium amount □ I've been doing this a lot
52. I've been expressing my negative feelings.
□ I haven't been doing this at all □ I've been doing this a little bit
□ I've been doing this a medium amount □ I've been doing this a lot
53. I've been trying to find comfort in my religion or spiritual beliefs.
□ I haven't been doing this at all □ I've been doing this a little bit
□ I've been doing this a medium amount □ I've been doing this a lot
54. I’ve been trying to get advice or help from other people about what to do.
□ I haven't been doing this at all □ I've been doing this a little bit
□ I've been doing this a medium amount □ I've been doing this a lot
55. I've been learning to live with it.
□ I haven't been doing this at all □ I've been doing this a little bit
□ I've been doing this a medium amount □ I've been doing this a lot
56. I've been thinking hard about what steps to take.
□ I haven't been doing this at all □ I've been doing this a little bit
□ I've been doing this a medium amount □ I've been doing this a lot
57. I’ve been blaming myself for things that happened.
□ I haven't been doing this at all □ I've been doing this a little bit
□ I've been doing this a medium amount □ I've been doing this a lot
58. I've been praying or meditating.

□ I haven't been doing this at all □ I've been doing this a little bit
□ I've been doing this a medium amount □ I've been doing this a lot

59. I've been making fun of the situation.
□ I haven't been doing this at all □ I've been doing this a little bit
□ I've been doing this a medium amount □ I've been doing this a lot

60. If you wish, you can share any short and relevant information related to your mental health, coping mechanisms and opportunities during CODIV-19

____

____

____

Data Protection

Disclaimer: The personally identifiable data that we are collecting through this form is collected only for medical research purposes, including its analysis and potential publishing. By providing your personal data through this form, you are providing your consent that your data is collected for the previously stated purposes to the EuroNET MPRH Project "COVID-19 related mental health, lost opportunities and coping mechanisms of Public Health Residents in Europe" researcher group. The use of the information provided in the form will be in line with the purpose of the form and will not be used otherwise. All data you enter is completely anonymous and no question can be used to identify you. The data will only be accessible by the creators and the researchers of this survey. If you would like to know more about how we manage your data, please email us at research@euronetmrph.org

□ I consent.
□ I do not consent to provide my data for this research project.
